# The benefits of contributing to the citizen science platform iNaturalist as an identifier

**DOI:** 10.1371/journal.pbio.3001843

**Published:** 2022-11-10

**Authors:** Corey T. Callaghan, Thomas Mesaglio, John S. Ascher, Thomas M. Brooks, Analyn A. Cabras, Mark Chandler, William K. Cornwell, Indiana Cristóbal Ríos-Málaver, Even Dankowicz, Naufal Urfi Dhiya’ulhaq, Richard A. Fuller, Carlos Galindo-Leal, Florencia Grattarola, Susan Hewitt, Lila Higgins, Colleen Hitchcock, Keng-Lou James Hung, Tony Iwane, Paula Kahumbu, Roger Kendrick, Samuel R. Kieschnick, Gernot Kunz, Chien C. Lee, Cheng-Tao Lin, Scott Loarie, Milton Norman Medina, Mark A. McGrouther, Lera Miles, Shaunak Modi, Katarzyna Nowak, Rahayu Oktaviani, Brian M. Waswala Olewe, James Pagé, Silviu Petrovan, cassi saari, Carrie E. Seltzer, Alexey P. Seregin, Jon J. Sullivan, Amila P. Sumanapala, Aristide Takoukam, Jane Widness, Keith Willmott, Wolfgang Wüster, Alison N. Young

**Affiliations:** 1 German Centre for Integrative Biodiversity Research (iDiv) Halle—Jena—Leipzig, Leipzig, Germany; 2 Institute of Biology, Martin Luther University Halle—Wittenberg, Halle (Saale), Germany; 3 Centre for Ecosystem Science; School of Biological, Earth and Environmental Sciences; UNSW Sydney; Sydney, Australia; 4 Evolution & Ecology Research Centre; School of Biological, Earth and Environmental Sciences; UNSW Sydney; Sydney, Australia; 5 Department of Biological Sciences, National University of Singapore, Singapore, Singapore; 6 International Union for Conservation of Nature (IUCN), Gland, Switzerland; 7 World Agroforestry Center (ICRAF), University of the Philippines, Los Baños, Philippines; 8 Institute for Marine & Antarctic Studies, University of Tasmania, Hobart, Tasmania, Australia; 9 Coleoptera Research Center, Institute of Biodiversity and Environment, University of Mindanao, Davao City, Philippines; 10 Earthwatch Institute, Boston, Massachusetts, United States of America; 11 McGuire Center for Lepidoptera and Biodiversity, Florida Museum of Natural History, University of Florida, Gainesville, Florida, United States of America; 12 Instituto de Investigaciones de Recursos Biológicos Alexander von Humboldt, Claustro de San Agustín, Villa de Leyva, Boyaca, Colombia; 13 Biology Department; Brandeis University; Waltham, Massachusetts, United States of America; 14 Independent Researcher, Mlati, Sleman, DI Yogyakarta, Indonesia; 15 School of Biological Sciences, The University of Queensland, Brisbane, Queensland, Australia; 16 Comisión Nacional para el Conocimiento y Uso de la Biodiversidad: Ciudad de Mexico, Ciudad de México, Mexico; 17 Faculty of Environmental Sciences, Czech University of Life Sciences Prague, Praha, Czech Republic; 18 Independent Researcher, New York, New York, United States of America; 19 Natural History Museum of Los Angeles County, Los Angeles, California, United States of America; 20 Oklahoma Biological Survey, University of Oklahoma, Norman, Oklahoma, United States of America; 21 iNaturalist, California Academy of Sciences, San Francisco, California, United States of America; 22 Wildlife Direct, Nairobi, Kenya; 23 Director, C & R Wildlife, Tai Po, Hong Kong; 24 Urban Wildlife Biologist, Texas Parks and Wildlife Department, Dallas, Texas, United States of America; 25 Karl Franzens University of Graz, Universitätsplatz 2, Department of Biology, Graz, Austria; 26 Institute of Biodiversity and Environmental Conservation, Universiti Malaysia Sarawak, Kota Samarahan, Sarawak, Malaysia; 27 Department of Biological Resources, National Chiayi University, Chiayi, Taiwan; 28 Senior Fellow, Australian Museum Research Institute, Australian Museum, Sydney, New South Wales, Australia; 29 UN Environment Programme World Conservation Monitoring Centre (UNEP-WCMC), Cambridge, United Kingdom; 30 Coastal Conservation Foundation, Matunga West, Mumbai, Maharashtra, India; 31 Faculty of Biology, University of Warsaw, Białowieża Geobotanical Station, Białowieża, Poland; 32 Yayasan Konservasi Ekosistem Alam Nusantara (KIARA), West Java, Indonesia; 33 Maasai Mara University, Narok, Kenya; 34 Baruk Yadiym Ecosphere, Nairobi, Kenya; 35 Kenya National Commission for UNESCO, Nairobi, Kenya; 36 Canadian Wildlife Federation, Kanata, Ontario, Canada; 37 Conservation Science Group, Department of Zoology, University of Cambridge, Cambridge, United Kingdom; 38 Chicago Park District, Chicago, Illinois, United States of America; 39 M.V. Lomonosov Moscow State University, Moscow, Russia; 40 Department of Pest-Management and Conservation, Lincoln University, New Zealand; 41 Department of Zoology and Environment Sciences, University of Colombo, Sri Lanka; 42 African Marine Mammal Conservation Organization (AMMCO), Kassala-Beach, Dizangue, Littoral, Cameroon; 43 Yale University Department of Anthropology, New Haven, Connecticut, United States of America; 44 Molecular Ecology and Fisheries Genetics Laboratory, School of Natural Sciences, Bangor University, Bangor, United Kingdom; 45 California Academy of Sciences, San Francisco, California, United States of America

## Abstract

As the number of observations submitted to the citizen science platform iNaturalist continues to grow, it is increasingly important that these observations can be identified to the finest taxonomic level, maximizing their value for biodiversity research. Here, we explore the benefits of acting as an identifier on iNaturalist.

In an increasingly human-modified world, biodiversity data are essential to the detection and understanding of local to global biodiversity change [1]. Biodiversity monitoring is targeted in the draft post-2020 global biodiversity framework of the Convention on Biological Diversity, recognizing that up-to-date knowledge is needed to guide decision-making. In recent decades, there has been a massive increase in available biodiversity data—there are currently >2.1 billion species occurrence records in the Global Biodiversity Information Facility, representing a 12-fold increase since 2007 [2]. This rise in biodiversity data is due in part to the growing popularity of citizen, or community-based, science.

One of the most globally successful platforms is iNaturalist (www.inaturalist.org; [3])—a multitaxa platform and joint initiative of the California Academy of Sciences and the National Geographic Society. iNaturalist allows participants to contribute observations of any organism (e.g., Fig 1), or traces thereof, along with associated spatiotemporal metadata. Observations are then identified and verified to high taxonomic resolution by the iNaturalist community, in conjunction with the rapidly improving computer vision suggestions. An observation is deemed “Research Grade” when it meets the site’s metadata quality criteria, and has 2 or more suggested identifications, more than two-thirds of which agree at a species level (i.e., 2/2, 2/3, 3/4, etc.; although records identified to a level finer than family can also become Research Grade if no further progress in identification is deemed possible). While the quantity of data and contributors continue to increase on iNaturalist, one bottleneck to fully realizing the potential of these data for scientific research is the dearth of participants with reasonable expertise (i.e., someone with the skills and ability to make informed identifications)—hereafter “identifiers”—actively participating in the community. The iNaturalist community—as of January 2022—consists of 2.5 million users, 92% of whom only observe, <1% of whom have only made identifications, and 7% of whom both observe and identify. More recruitment of identifiers is clearly needed. Here, we provide our collective perspective on 7 reasons to contribute to iNaturalist as an identifier (Box 1).

**Fig 1:**
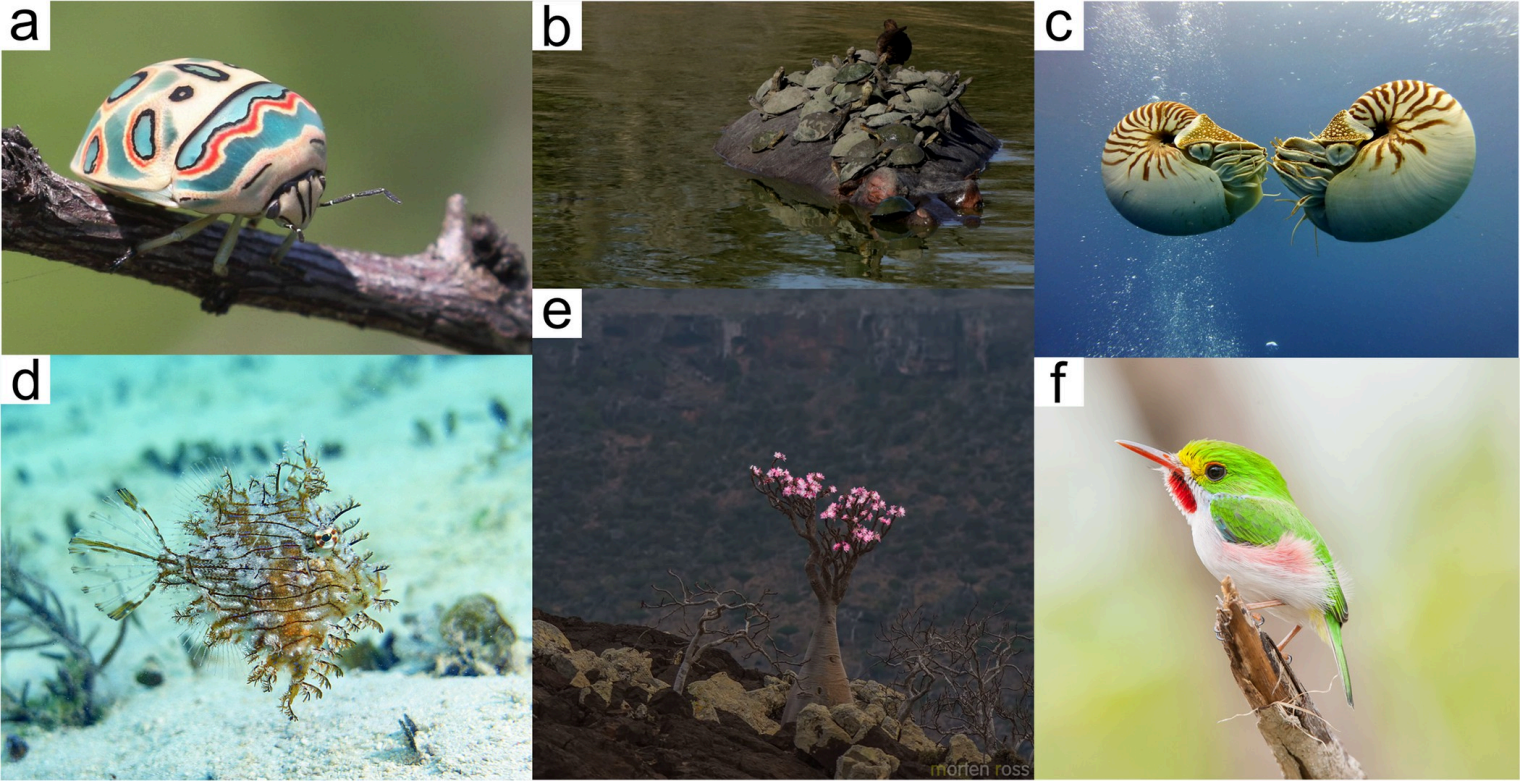
Selection of iNaturalist’s most favorited observations. (**a**) Picasso Bug (*Sphaerocoris annulus*), Alan Manson (@alandmanson); (**b**) South African Hippopotamus (*Hippopotamus amphibius* ssp. *capensis*) ferrying Helmeted Turtles (*Pelomedusa subrufa*), Serrated Hinged Terrapins (*Pelusios sinuatus*), and a Hamerkop (*Scopus umbretta*), Maritza de Kock (@maritzasouthafrica); (**c**) Palau Nautilus (*Nautilus belauensis*), Kai Squires (@squiresk); (**d**) juvenile Tasselled Leatherjacket (*Chaetodermis penicilligerus*), Glen Whisson (@glen_whisson); (**e**) Socotran Desert Rose (*Adenium obesum* ssp. *sokotranum*), Morten Ross (@morten); (**f**) Cuban Tody (*Todus multicolor*), Wayne Fidler (@wayne_fidler).

**Box 1. Seven reasons to contribute to iNaturalist as an identifier**

**1. Your contributions increase knowledge of biodiversity**

- When you add an identification to an observation, it can immediately increase the value of that record by advancing the taxonomic level to which that observation is identified.
- Identification efforts can be prioritized for maximum knowledge increase (e.g., by identifying species in undersampled regions of the world, targeting specific taxonomic groups that are threatened, or focusing on regions of the world with high endemism).

**2. The value of opportunistic records is increasing**

- As a result of rapidly increasing statistical advances and data integration approaches with structured sampling, each identified record can advance our understanding of species distributions and abundance trajectories.
- Photographs from iNaturalist are increasingly used in many unique and novel secondary ways, often opportunistic in nature.

**3. You can contribute data on threatened, data-deficient, or invasive species**

- Since its inception, iNaturalist users have documented many significant records, including the rediscovery of species thought to be extinct or locally extirpated, considerable range extensions and new national records, previously undocumented behaviors and host associations, and even the discovery and subsequent descriptions of new species.
- iNaturalist is useful in monitoring pathognomonic spread to new locations and for rapid responses in detecting novel introductions.

**4. iNaturalist is a ready-made, free, and easy-to-use data collection infrastructure**

- A computer or smartphone and an internet connection are the only requirements for using iNaturalist, with all aspects of the platform, including uploading, identifying, and downloading data, entirely free.
- An important component of the iNaturalist infrastructure is the computer vision providing automated identification suggestions.
- iNaturalist features a dedicated “Identify: tool (www.inaturalist.org/observations/identify) that is streamlined for a rapid workflow to make, and review, identifications quickly.

**5. You can partake in dynamic, real-time interactions around the world**

- Engaging with iNaturalist prompts you to discuss and collaborate with all types of users in real time, with benefits for everyone involved.
- Discussing identifications is a way of honing and expanding your own skills, including the opportunity for more experienced experts to validate the identifications of less-experienced experts, training the next generation of identifiers.

**6. You can engage with a broader audience**

- iNaturalist offers an efficient and powerful mechanism for broader societal impact, since identifiers can engage with thousands of individuals around the world, helping to connect people with the ecosystems of which they are a part.
- Engaging with participants through the platform can also improve the quality and quantity of observations that are useful for biodiversity science.

**7. You can enjoy yourself**

- Browsing photographs of even well-known species, and helping new naturalists to identify them, can be enjoyable and personally rewarding.
- There is an official “iNat Observation of the Day” project (see here) showcasing such observations.

Non-English translations of this manuscript are available here.

## How identifications can contribute to biodiversity research

As an identifier on iNaturalist, you can identify observations that have been made anywhere in the world, and your identification efforts can be prioritized for maximum knowledge increase. For example, correct identifications of poorly studied invertebrates from the tropics are arguably more valuable than identifications of common birds in the United States. Past misidentifications can be transparently amended on iNaturalist, and the person who made the observation, and any potential future identifiers, can learn about the identification of that organism. Even if an observation is not identifiable to the species level, an expert identification to family or genus, coupled with teaching the observer what to look for and capture next time (i.e., photographing particular attributes of organisms), is often useful for improving data quality in the future.

In addition to the use of opportunistic records to quantify biodiversity in space or time, photographs from iNaturalist are also being used in many unique and novel secondary ways, often opportunistic in nature. For example, opportunistic records have been used to study timing of winter coat molt in mountain goats [4]. The usability of a photograph for these purposes is most valuable when the observation has been identified to the finest possible taxonomic resolution, something that increasing the number of expert identifiers can help achieve. Identifiers can further add value to observations with iNaturalist annotations (e.g., Plant Phenology = Flowering), observation fields (e.g., Host Species), and adding observations to projects (e.g., bees concentrating nectar; [5]).

Since its inception, iNaturalist users have documented many significant records, including the rediscovery of species thought to be extinct or locally extirpated (e.g., [6]) considerable range extensions and new national records (e.g., [7]), and previously undocumented behaviors and host associations (e.g., [8]). Observations uploaded to the project *Australasian Fishes* have contributed more than 600 novel findings of undescribed species, range extensions, and undocumented behaviors and species interactions (e.g., [9]), while the project *First Known Photographs of Living Specimens* has more than 3,600 records representing the first, and often only, photographic records of those taxa [10]. Novel observations identified promptly can be especially important from a biosecurity perspective. Citizen science can enable the early detection of invasive species [11], and, indeed, there are an increasing number of records of invasive species having been first detected via iNaturalist. On 31 July 2020, a photograph of feeding damage on an elm leaf (*Ulmus* sp.) was uploaded to iNaturalist (see here) by user Alain Hogue (@alainhogue). Within 8 hours, Charley Eiseman (@ceiseman), a North American expert in leaf mining and other herbivorous insect tracks and signs, suggested that the observation may represent the first North American record for the Elm Zig-zag Sawfly (*Aproceros leucopoda*), an invasive pest species outside its native range in eastern Asia. The observation sparked onsite visits by the Canadian Food Inspection Agency and Canadian Forest Services, where specimens were collected, and additional searches of *Ulmus* observations from Canada and the USA uncovered more records [12].

A consistent thread across many of these “special” records is that, until seen and identified by an expert, they are just another record among the millions uploaded to the platform. Indeed, there are likely thousands of range shifts or extensions, new national records, rediscoveries, or newly introduced species that have been uploaded to iNaturalist but gone unnoticed due to a dearth, or even absence, of identifiers for particular taxa or regions.

## Conclusions

iNaturalist is revolutionizing our understanding of biodiversity at multiple spatial and temporal scales and across society. While we focus on individuals dedicating their own time identifying iNaturalist observations, we recognize that institutional support for experts to dedicate time to identify observations is an additional opportunity that would yield similar benefits. In the same vein, there exist barriers to the widespread use of iNaturalist globally (i.e., access to the internet or smartphones) that will need to be fully overcame to maximize the value of iNaturalist for biodiversity research in the future. Nevertheless, in the short term, we hope that you will consider contributing your expertise to iNaturalist—a time investment of 30 minutes per day, week, or month can provide substantial contributions to collectively improving our understanding of biodiversity. We conclude by offering an open invitation to all prospective identifiers to reach out to us on iNaturalist for any advice or guidance (see S1 Table).

## Supporting information

S1 Table A table of the authors’ iNaturalist handles for those interested in reaching out to the authors on the iNaturalist platform. (DOCX)
