# Supplementary material for: The benefits of contributing to the citizen science platform iNaturalist as an identifier
Source: PLoS Biol. 2022 Nov 10;20(11):e3001843. doi: 10.1371/journal.pbio.3001843 (PMC9648699; doi:10.1371/journal.pbio.3001843)
Supplement: S1 Table — (DOCX) [file pbio.3001843.s001.docx]

**S1 Table**. We actively encourage anyone interested in joining iNaturalist to reach out to us, and here are the iNaturalist usernames for each of the co-authors.

| **Name** | **iNaturalist username** |
| --- | --- |
| Corey T. Callaghan | @coreytcallaghan |
| Thomas Mesaglio | @thebeachcomber |
| John S. Ascher | @johnascher |
| Thomas M. Brooks | @tbrooks |
| Analyn A. Cabras | @anncabras24 |
| Mark Chandler | @markchandler |
| William K. Cornwell | @wcornwell |
| Indiana Cristóbal Ríos-Málaver | @indianacristo |
| Even Dankowicz | @edanko |
| Naufal Urfi Dhiya’ulhaq | @naufalurfi |
| Richard A. Fuller | @rich_fuller |
| Carlos Galindo-Leal | @carlos2 |
| Florencia Grattarola | @flo_grattarola |
| Susan Hewitt | @susanhewitt |
| Lila Higgins | @lhiggins |
| Colleen Hitchcock | @hitchco |
| Keng-Lou James Hung | @kjhung |
| Tony Iwane | @tiwane |
| Paula Kahumbu | @paulakahumbu |
| Roger Kendrick | @hkmoths |
| Samuel R. Kieschnick | @sambiology |
| Gernot Kunz | @gernot2 |
| Chien C. Lee | @cclborneo |
| Cheng-Tao Lin | @mutolisp |
| Scott Loarie | @loarie |
| Milton Norman Medina | @mnmedinaphilippines |
| Mark A. McGrouther | @markmcg |
| Lera Miles | @lera |
| Shaunak Modi | @shaunak |
| Katarzyna Nowak | @katzyna |
| Rahayu Oktaviani | @roktaviani |
| Brian M. Waswala Olewe | @waswala |
| James Pagé | @jpage_cwf |
| Silviu Petrovan | @silviupetrovan |
| cassi saari | @bouteloua |
| Carrie E. Seltzer | @carrieseltzer |
| Alexey P. Seregin | @apseregin |
| Jon J. Sullivan | @jon_sullivan |
| Amila P. Sumanapala | @amila_sumanapala |
| Aristide Takoukam | @aristidetakoukam |
| Jane Widness | @jwidness |
| Keith Willmott | @kwillmott |
| Wolfgang Wüster | @wolfgang_wuster |
| Alison N. Young | @kestrel |
